# Common synaptic inputs and persistent inward currents of vastus lateralis motor units are reduced in older male adults

**DOI:** 10.1007/s11357-024-01063-w

**Published:** 2024-01-19

**Authors:** Yuxiao Guo, Eleanor J. Jones, Jakob Škarabot, Thomas B. Inns, Bethan E. Phillips, Philip J. Atherton, Mathew Piasecki

**Affiliations:** 1 Centre of Metabolism, Ageing & Physiology (COMAP), MRC-Versus Arthritis Centre for Musculoskeletal Ageing Research &, National Institute for Health Research (NIHR) Nottingham Biomedical Research Centre, School of Medicine, University of Nottingham, Royal Derby Hospital Centre (Room 3011), Derby, DE22 3DT UK; 2 School of Sport, Exercise and Health Sciences, Loughborough University, Loughborough, UK

**Keywords:** Ageing, Motor unit, Persistent inward current, Common synaptic input, Vastus lateralis

## Abstract

Although muscle atrophy may partially account for age-related strength decline, it is further influenced by alterations of neural input to muscle. Persistent inward currents (PIC) and the level of common synaptic inputs to motoneurons influence neuromuscular function. However, these have not yet been described in the aged human quadriceps. High-density surface electromyography (HDsEMG) signals were collected from the vastus lateralis of 15 young (mean ± SD, 23 ± 5 y) and 15 older (67 ± 9 y) men during submaximal sustained and 20-s ramped contractions. HDsEMG signals were decomposed to identify individual motor unit discharges, from which PIC amplitude and intramuscular coherence were estimated. Older participants produced significantly lower knee extensor torque (*p* < 0.001) and poorer force tracking ability (*p* < 0.001) than young. Older participants also had lower PIC amplitude (*p* = 0.001) and coherence estimates in the alpha frequency band (*p* < 0.001) during ramp contractions when compared to young. Persistent inward currents and common synaptic inputs are lower in the vastus lateralis of older males when compared to young. These data highlight altered neural input to the clinically and functionally important quadriceps, further underpinning age-related loss of function which may occur independently of the loss of muscle mass.

## Introduction

Human ageing is characterised by a progressive impairment of muscle force generation, control, and overall physical performance [1–3], all of which can negatively impact quality of life [4, 5]. The loss of muscle size is explicable by the combined effects of muscle fibre atrophy and muscle fibre loss [2], and although atrophy may partially account for declines in muscle strength, it is further influenced by structural and functional alterations of the nervous system.

The voluntary contraction of muscle fibres occurs via net excitatory signals to motor units (MUs); groups of muscle fibres innervated by a single motoneuron [6]. The central nervous system controls muscle force via two primary strategies; varying the number of MUs recruited and varying the discharge rate of each motor neuron, both of which are susceptible to age-related alterations. Substantial MU loss occurs into the seventh decade [7] alongside the compensatory process of MU remodelling, culminating in MUs that are fewer in number and larger in innervation ratio (fibre number) [8–11]. Human spinal cord sections showed an approximate 50% loss of motoneurons in individuals above 60 years of age [12], while EMG methods estimated around 40% fewer MUs in old compared to young VL [13]. These findings are supported in greater detail in animal models [14]. The discharge rate of MUs is also commonly reported to be reduced with age when assessed at normalised contraction intensities. In the VL this was associated with reduced sex hormone concentrations [15] suggesting a possible systemic effect, yet meta-analysis data shows the age-related reduction is not applicable to all muscles [16].

Motoneuron activation is regulated by two fundamental mechanisms: ionotropic input and neuromodulation [17]. Ionotropic input entails the direct release of neurotransmitters onto post-synaptic ligand-gated channels within the spinal cord, leading to the depolarisation or hyperpolarisation of motoneurons. Neuromodulation operates via intracellular second messenger systems, which modulate the voltage- and ligand-gated channels of neurons. Essentially, neuromodulation modifies the response of motoneurons to ionotropic input, eliciting changes in their functional characteristics [18, 19]. This process is enhanced by monoamines such as serotonin and norepinephrine, which are released from the brainstem nuclei. These monoamines stimulate inward-flowing persistent calcium and sodium currents [17], known as persistent inward currents (PICs). As long as the membrane potential remains above the activation threshold, these PICs tend to remain activated [17], combined with neuromodulatory drive amplifying the firing behaviours of motoneurons to synaptic inputs up to five-fold [20] as well as generating long-lasting plateau potentials which results in self-sustaining firing [21]. Age-related changes in neuromodulation, specifically the modulation of motoneuron activation, have been documented such as decreased effectiveness of neuromodulatory systems, impaired second messenger system, and alterations in PIC activation which can contribute to alterations in motor function during the ageing process. Estimates of PIC magnitude decrease with age in both upper [22] and lower limb muscles [23], and are also responsive to resistance exercise intervention [24, 25].

Rather than controlling the discharge pattern of individual MUs, the central nervous system controls the excitatory inputs to the MU pool, referred to as common synaptic inputs [26] which are defined as the proportion of net synaptic input correlated between MUs. Force fluctuations are largely determined by the alterations in low-frequency components of common synaptic inputs to MUs, which is reflected in concurrent fluctuations in MU discharge rates from the same MU pool [27]. Accordingly, a decrease in force steadiness associated with ageing may be a consequence of an increase in the variance of common synaptic inputs [28, 29]. However, the variability of common synaptic inputs in quadriceps has only been explored in healthy young individuals [30, 31], and as a muscle that is susceptible to age-related loss of mass and function [32, 33] data from older people is warranted.

It is not possible to make intracellular recordings from human spinal motoneurons in order to estimate PICs, so alternative methods are employed which distinguish the intrinsic excitability of the MU from its descending synaptic drive by using the unit-wise MU analysis technique [22]. In view of the advances with which MU discharging patterns can be measured, it provides an opportunity to investigate and comprehend the physical properties and underlying mechanisms which may impair motor control in older age. Therefore, the aim of this study was to explore the differences in physical performance as well as the magnitude of persistent inward currents and common synaptic inputs in the vastus lateralis between healthy young and older males. We hypothesised that older participants would exhibit greater physical decrements and lower estimates of persistent inward currents and common synaptic inputs.

## Methods

### Participants and ethical approval

This study was approved by the University of Nottingham Faculty of Medicine and Health Science Research Ethics Committee (90–0820, 199–0221) and conformed to the Declaration of Helsinki.

Fifteen healthy young males between the ages of 18–40 (mean ± SD; age: 23.1 ± 5.2 years; body mass index: 25.0 ± 2.5 kg/m^2^) and 15 healthy older males between the ages of 55 to 85 (67.2 ± 8.9 years; 26.1 ± 2.3 kg/m^2^) provided informed consent to take part in the study. All participants were recruited through advertisements in the local community. Prior to enrolment, all participants completed a comprehensive clinical examination, and metabolic screening was conducted at the School of Medicine, Royal Derby Hospital Centre. All recruited participants were recreationally active and would be excluded if they displayed evidence of BMI < 18.5 or > 35 kg/m^2^, were competitive in an athletic discipline at the county level or above, and had musculoskeletal disorders, respiratory disease, neurological disorders, metabolic disease, active cardiovascular problems, active inflammatory bowel or renal disease, recent steroid treatment within 6 months or hormone replacement therapy, and family history of early (< 55 years) death from cardiovascular disease. Participants who were currently using beta-blockers or selective serotonin reuptake inhibitors (SSRIs) were also excluded. Among all the participants, two individuals in the older group exhibited left dominance, while the remaining participants demonstrated right dominance.

### Anthropometry

Ultrasound was used to measure the cross-sectional area (CSA) of the VL muscle of the right leg, using an ultrasound probe (LA523 probe, B-mode, frequency range 26–32 Hz, and MyLabTM50 scanner, Esaote, Genoa, Italy) at the anatomical mid-point of the right thigh. Images were recorded with the participants laying in a supine position, and the mid-point was obtained from the identification of the greater trochanter at the top of the femur and the mid-point of the patella. Panoramic imaging (VPAN) images were obtained in a medial-to-lateral fashion, beginning and ending the capture of the image at the aponeurosis borders of the VL of the right leg. The produced images were subsequently analysed using ImageJ software (National Institute of Health, USA) through tracing around the contour of the aponeurosis via polygon function to quantify the area of the VL. Each image was analysed three times and the mean value of three images was accepted as CSA.

### Experimental protocol

All participants were required to attend the lab at 0900 after an overnight fast and abstain from strenuous exercise 24 h prior to the testing sessions. Participants were seated in a custom-built chair with hips and knees flexed at ~ 90°. The lower leg was secured to a force dynamometer with noncompliant straps above the medial malleolus. To minimise the movement of the upper trunk during testing, a seat belt was fastened across the pelvis of the participant. After a standardised warm-up through several submaximal contractions [34], participants were instructed to contract as hard and fast as they could. During the trial, participants were not allowed to hold onto the side of the chair and were asked to cross their arms across the chest. This was performed with real-time visual force feedback on a monitor placed in front of the participant, with verbal encouragement to aid in producing maximal effort. This was repeated two to three times further, giving 60 s of rest between attempts of standardised recovery. If the difference between the last two attempts was < 5%, the highest value recorded in newtons was taken and accepted as the maximal isometric voluntary contraction force (MVC). Muscle torque was calculated by multiplying MVC by the lever arm length from the knee to the centre of the ankle strap.

Following a 60-s rest, participants were then asked to perform four submaximal voluntary isometric contractions at 25% of MVC (~ 12 s) and four triangular-shaped contractions (10-s up and 10-s down) peaking at 20% of MVC, with 30-s intervals between contractions. In each case, a target line was displayed on a screen and participants were instructed to follow the target as close as possible. Ramp contractions to 20% of MVC have been widely used for estimating persistent inward currents (details below) [21, 35, 36]. To quantify force steadiness during 25% of MVC, the coefficient of variation of the force (CoV) was calculated = (SD/Mean)*100. To assess force tracking accuracy, all data were exported to Spike2 (version 9.11, CED Ltd., Cambridge, UK), where a virtual channel was created by subtracting the performed path from the requested (target) path and rectifying it. The area under the curve reflects the level of deviation from the target line reflecting muscle force tracking accuracy, with higher values indicating greater deviation.

### High-density surface electromyography

A semi-disposable high-density surface electromyography (HDsEMG) array (64 electrodes, 13 × 5, 8 mm, I.E.D., GR08MM1305, OT Bioelettronica, Inc., Turin, Italy) was placed with the orientation of the muscle fibres (proximal to distal) over the muscle belly of right vastus lateralis after skin preparation involving shaving, light abrasion and cleansing with 70% ethanol. Electrodes were secured to the skin using flexible tape. The adhesive grids were attached to the surface of the muscle by disposable bi-adhesive foam layers (SpesMedica, Bettipaglia, Italy). The skin electrode contact was facilitated by filling the cavities of the adhesive layers with conductive paste (AC Cream, SpesMedica). A strap ground electrode (WS2, OTBioelettronica, Turin, Italy) dampened with water was positioned around the ankle of the right leg to optimise the signal quality. HDsEMG signals were acquired in a monopolar configuration, amplified (× 256) with filtering set at 10–500 Hz and digitally converted at 2048 Hz by a 16-bit wireless amplifier (Sessantaquattro, OTBioelettronica, Turin, Italy) and transferred to a PC for further offline analysis. HDsEMG signals were recorded in OTBioLab software (OT Bioelecttronica, Turin, Italy).

The recorded HDsEMG signals were converted into a MatLab file comprising a single contraction. Each participant performed four submaximal contractions and ramp contractions; however, a single trial for each contraction protocol was selected for analysis based on the smoothness of the force profile. Monopolar HDsEMG signals were band-pass filtered at 20–500 Hz and then decomposed offline into MU pulse trains by a convolutive kernel compensation algorithm [37, 38]. After the decomposition, a trained investigator manually inspected the MU spike trains and edited the discharge patterns of each MU detected. Only MUs with a pulse-to-noise ratio equal or greater than 30 dB were kept for further analysis.

The mean discharge rate during the submaximal contraction at 25% of MVC was obtained and discharge rate variability was reported as the coefficient of variation (CoV) of the interspike interval (ISI) displayed as a percentage. Peak discharge rates during a ramp contraction were measured as the highest value of the smoothed MU discharge rate with a fifth-order polynomial.

### Estimation of persistent inward currents (PICs)

**Fig. 1:**
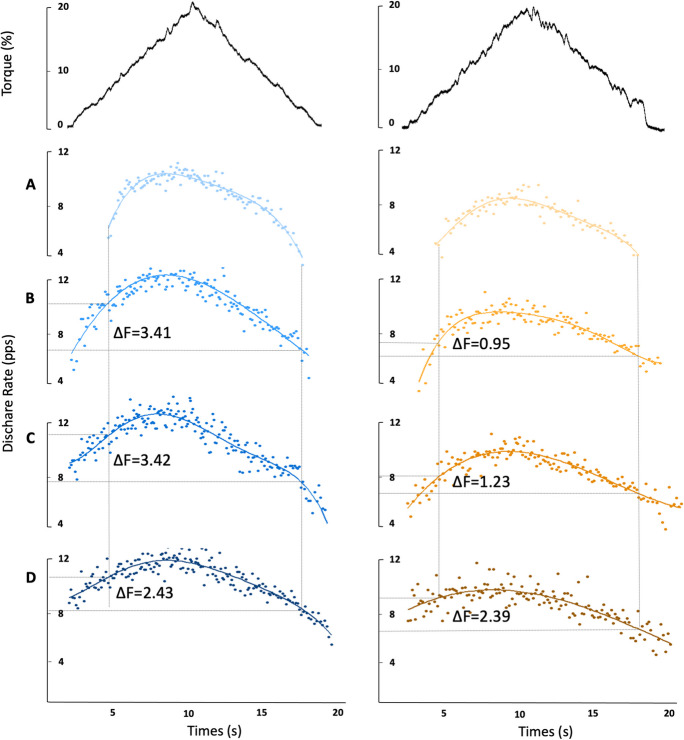
Example data showing *ΔF* calculation in vastus lateralis for paired-motor unit technique. Paired motor unit data from a young male is displayed in the left panels (mean *ΔF* = 3.09) and from an older male in the right panels (mean *ΔF* = 1.52). Test units are displayed in (**A**) and corresponding control units are in (**B**, **C**, and **D**)

The instantaneous firing rates of both MUs were calculated as the inverse of the interspike intervals of each MU spike train and smoothed by fitting a fifth-order polynomial function. An estimate of the PIC magnitude was derived using the unit-wise MU analysis technique [22]. The lower threshold unit to be recruited in the ramp contraction is commonly referred to as the “control” unit; the “test” unit is a unit of higher threshold. All MUs from 10 young and 10 older participants were initially isolated but those that met the criteria below were eventually included for *ΔF* (DeltaF) calculation. *ΔF*, the contribution of persistent inward currents to self-sustained firing, was calculated as the difference in “control” unit firing rate between the onset and offset of a “test” unit [21]. This technique relies on several assumptions: (1) test and control units share the common synaptic drive; (2) PIC is activated before or at the recruitment; (3) the firing rate of the control MU closely reflects the depolarizing input to the parent motoneuron; and (4) test and control unit process the synaptic inputs in a similar way. The MU pairs for the *ΔF* calculation were selected based on the following criteria: (1) as a measure of common synaptic modulation, rate-to-rate correlation *r* ≥ 0.7; (2) to avoid the high variability in *ΔF* calculation due to the initial acceleration phase of MU firings, test units were recruited at least 1 s after the control units; and (3) to account for the possibility of control unit saturation leading to an underestimation of *ΔF*, pairs in which rate modulation of the control unit fell within 0.5 pps were removed from analysis [21, 36, 39, 40]. An average *ΔF* was calculated when individual test units were paired with multiple control units (Fig. 1).

### Coherence estimates

**Fig. 2:**
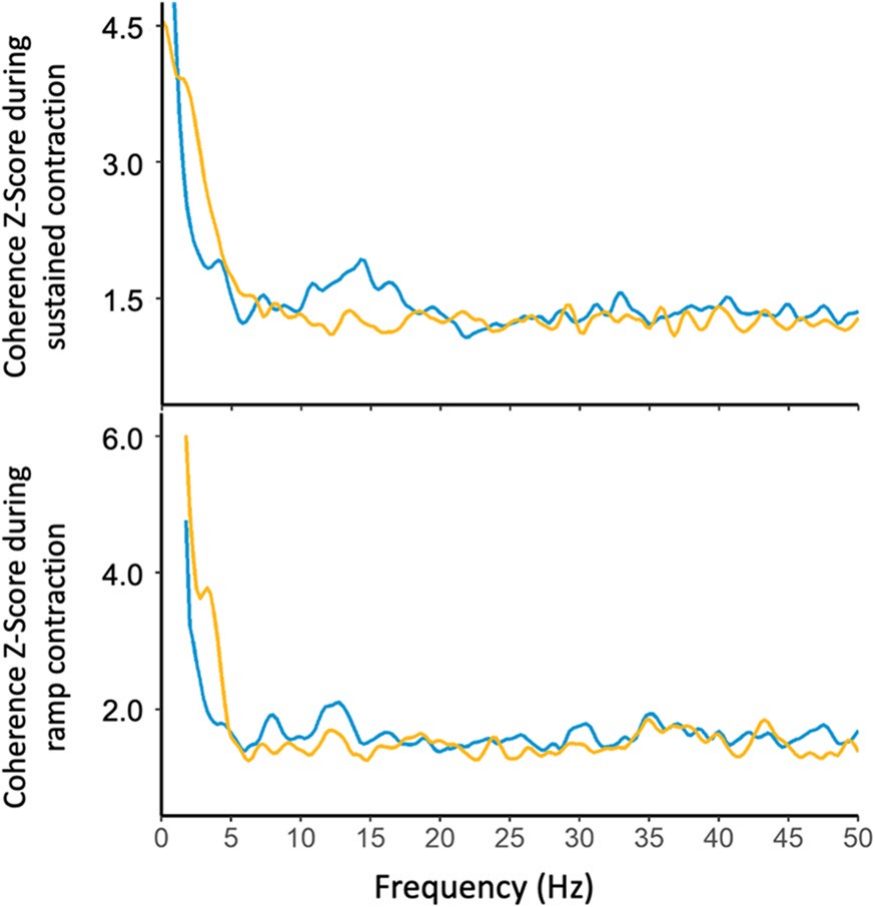
The group mean intramuscular coherence Z-scores during submaximal contraction at 25% of maximal isometric voluntary contraction (MVC) and ramp contraction at 20% of MVC in young (blue) and older (yellow) participants. The magnitude-squared coherence was calculated from all the combinations (a maximum of 60 random permutations) of two equally sized cumulative spike trains, each composed of three motor units

To estimate the level of common synaptic input, intramuscular coherence was calculated separately during sustained and ramp contractions (Fig. 2) and represents a frequency domain correlation between cumulative spike trains (CSTs) of the identified motor units. The magnitude-squared coherence was calculated using the Welch’s averaged periodogram with 50% overlapping Hann windows of 1 s at the different frequency bands: delta (0–5 Hz), alpha (5–12 Hz), beta (15–30 Hz), and piper (40–50 Hz) [41, 42]. This analysis was repeated for 60 randomly chosen combinations of two equally sized CSTs calculated as the sum of firing times from three MUs randomly selected from the same set of MUs and then averaged. A standard Z-transform was applied to the coherence estimates to obtain a normally distributed variable for comparisons [31, 43].

### Statistical analysis

Data management and analysis were performed using RStudio (Version 2022.07.1). Descriptive data were generated for all variables. An independent *t*-test was conducted to test whether any differences existed between young and older males in muscle cross-sectional area, torque, CoV-force, force tracking accuracy, and coherence estimates. In order to preserve variability within and across participants simultaneously to the greatest extent, multilevel linear regression models were generated to compare mean discharge rate, discharge rate variability, peak discharge rate, *ΔF*, recruitment threshold, and derecruitment threshold between groups using lme4 package (version 1.1–27.1) [44]. In the multilevel models, a single MU was regarded as the first level; and individual participant with clustered MUs was considered as the second level. For data visualization, individual participant means are displayed in box-and-jitter plots. A *p* value < 0.05 was considered statistically significant.

## Results

**Fig. 3:**
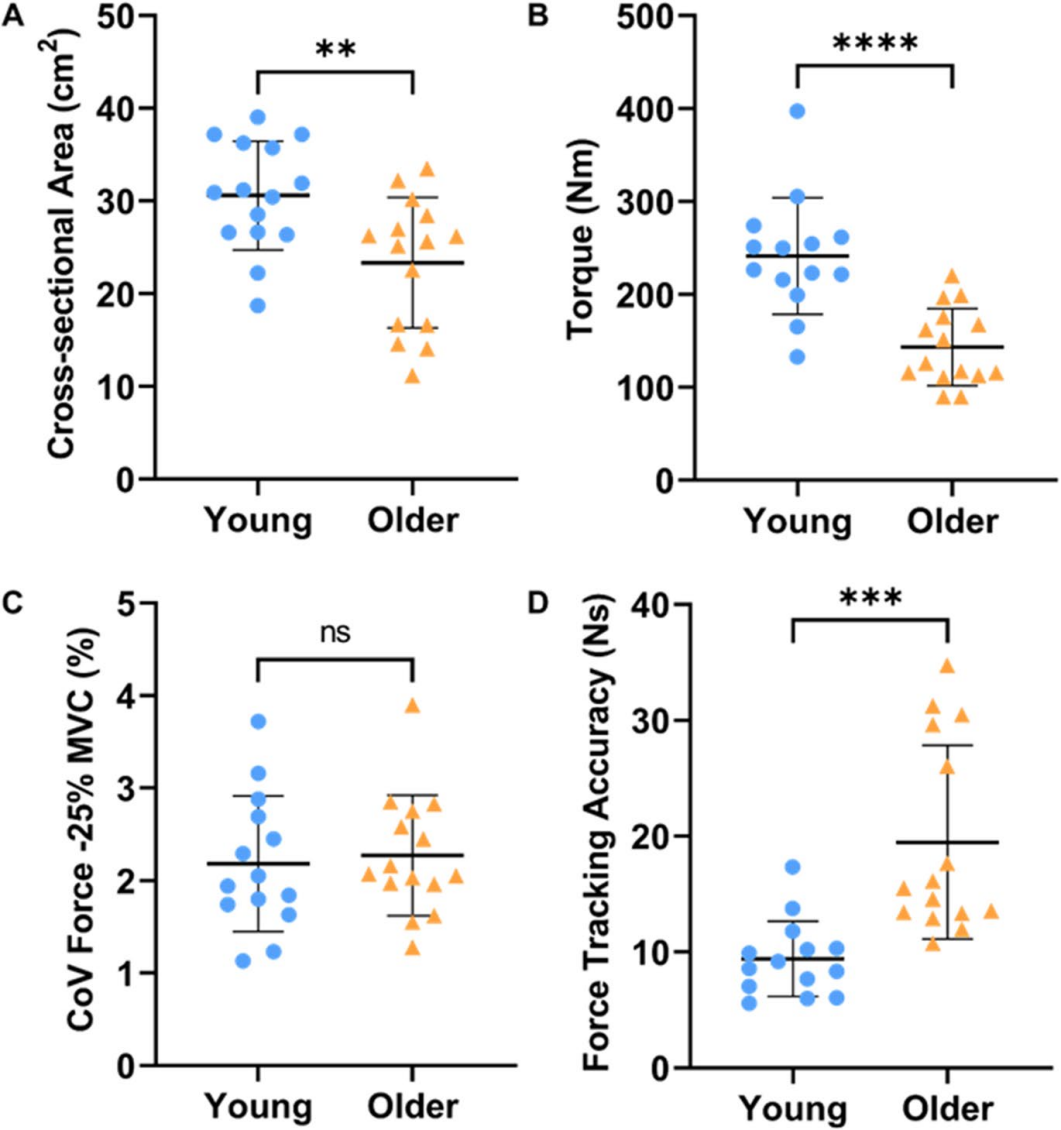
Cross-sectional area (**A**), torque (**B**), coefficient of variance of force at 25% maximal voluntary contraction (**C**), and force tracking accuracy (area under curve) during a single ramp contraction at 20% of maximal voluntary contraction (**D**) between young (blue) and older (yellow) males. Lines indicate group means and SD. ***p < 0.001, **p < 0.010, *p < 0.05

Young males had a larger muscle cross-sectional area (Y vs O: 30.59 ± 5.87 vs 23.34 ± 7.03 cm^2^, *p* = 0.005; Fig. 3A) and greater muscle torque (241.2 ± 62.69 vs 143.3 ± 41.50 Nm, *p* < 0.0001; Fig. 3B) than older males. There was no difference in force steadiness (CoV-Force) (2.18 ± 0.73 vs 2.27 ± 0.65%, *p* = 0.735; Fig. 3C) during sustained contractions. However, there was a significant difference between groups in force tracking accuracy of the ramp contraction, with the younger group performing better than the old (9.41 ± 3.25 vs 19.47 ± 8.36 Ns, *p* < 0.001; Fig. 3D).

**Fig. 4:**
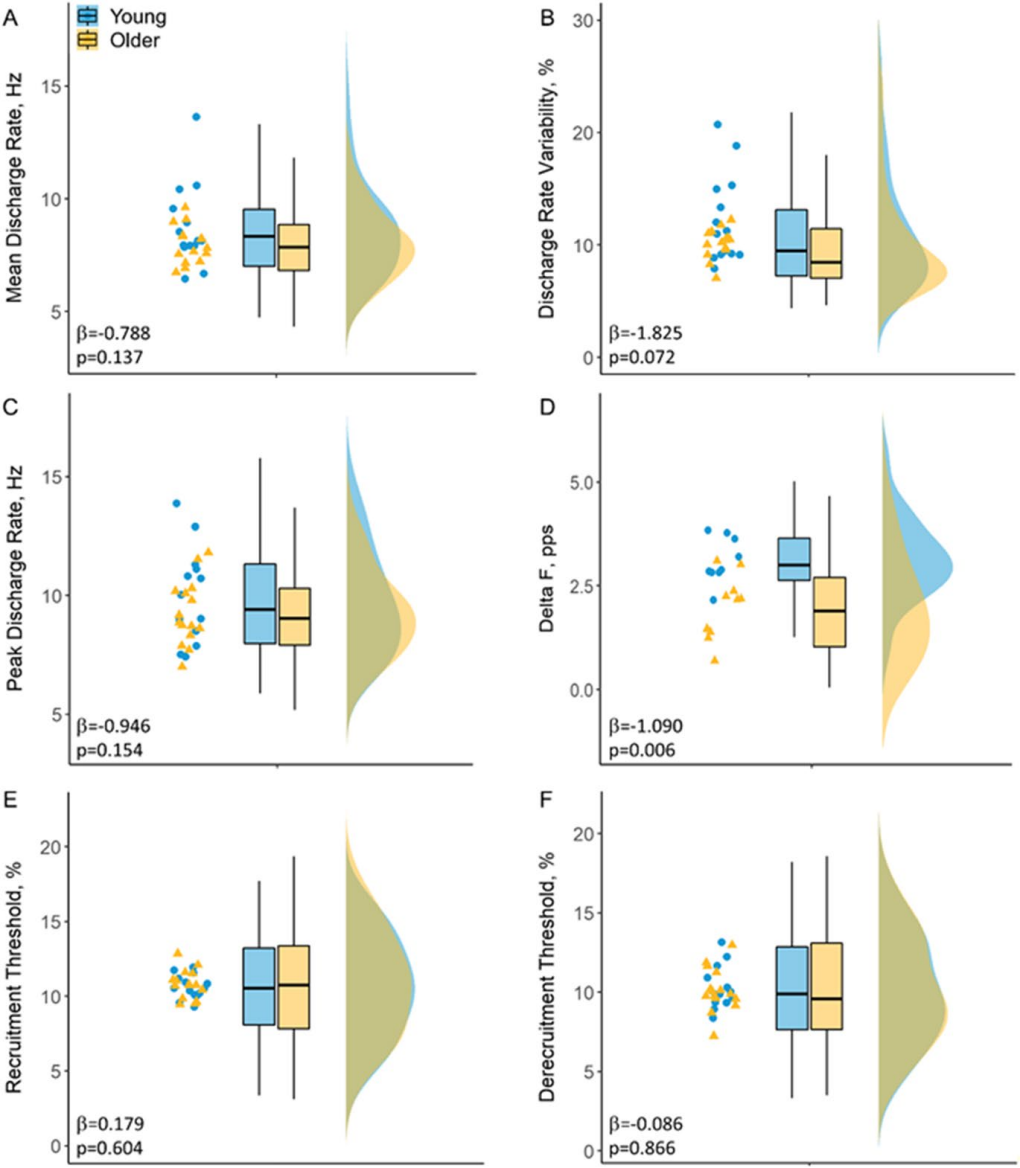
Jitter plots of the individual participant mean values and box and raincloud plots of mean discharge rate (**A**), discharge rate variability (**B**), peak discharge rate (**C**), *ΔF* (**D**), and recruitment/derecruitment threshold (**E** and **F**) in young (blue) and older (yellow) males

During the submaximal contraction at 25% of MVC, there was no statistical difference between groups in the number of MUs identified (*p* = 0.674), with a total of 246 MUs identified from young males (18 ± 10 per contraction per individual) and 282 MUs from older males (19 ± 5). There was no statistical difference in mean discharge rates (8.50 ± 2.08 vs 7.98 ± 1.64 pps, *p* = 0.137, Fig. 4A) or discharge rate variability (11.34 ± 6.01 vs 10.22 ± 5.15%, *p* = 0.072, Fig. 4B) between groups.

During the ramp contractions peaking at 20% of MVC, a total of 256 MUs were identified from young males (18 ± 9 per contraction per individual) and 262 MUs from older males (17 ± 3). There was no statistical difference in peak discharge rates (9.63 ± 2.20 vs 9.35 ± 1.94 pps, *p* = 0.154; Fig. 4C). For *ΔF* calculations, there was an average of 5 (SD 4) pairs of test and control units per person in the young and 6 (5) pairs in the older group, with the mean number of 4 (2) test units per person in young and 3 (2) in old. Younger males had significantly greater *ΔF* values when compared to older participants (3.11 ± 0.77 vs 1.86 ± 0.65 pps, *p* = 0.006; Fig. 4D). There was no significant difference in recruitment (10.60 ± 3.37 vs 10.75 ± 3.55%, *p* = 0.604; Fig. 4E) or derecruitment thresholds (10.14 ± 3.40 vs 10.18 ± 3.39%, *p* = 0.866; Fig. 4F) of the identified MUs between young and older groups.

**Fig. 5:**
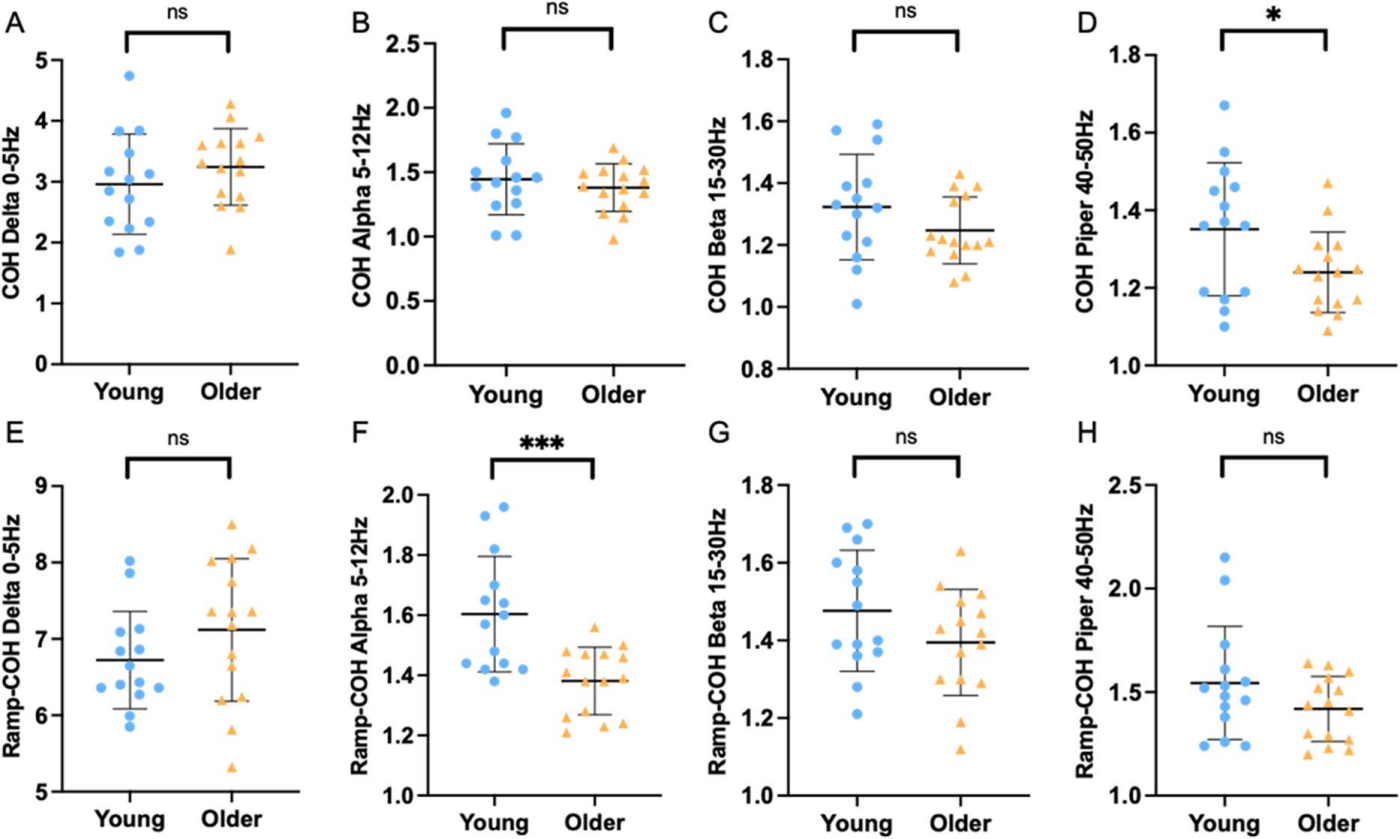
Individual participant means of intramuscular coherence Z-score at different frequency bands during a submaximal contraction at 25% maximal voluntary contraction (MVC) and a ramp contraction at 20% MVC in young (blue) and older (yellow) males

In the sustained contraction, there was no significant age-related difference in coherence in delta (2.96 ± 0.82 vs 3.24 ± 0.63, *p* = 0.305), Alpha (1.45 ± 0.28 vs 1.38 ± 0.19, *p* = 0.459) or beta (1.32 ± 0.17 vs 1.25 ± 0.11, *p* = 0.163) bands; however, the young had higher coherence in the piper band (1.35 ± 0.17 vs 1.24 ± 0.10, *p* = 0.042) (Fig. 5A–D). In the ramped contraction, there were no age-related differences in COH delta (6.72 ± 0.64 vs 7.12 ± 0.93, *p* = 0.196), beta (1.48 ± 0.16 vs 1.40 ± 0.14, *p* = 0.144) or piper (1.54 ± 0.27 vs 1.42 ± 0.16, *p* = 0.137) bands; however, young participants had greater coherence estimates in Alpha band (1.60 ± 0.19 vs 1.38 ± 0.11, *p* < 0.001) (Fig. 5E–H).

## Discussion

Here, we provide the first evidence for reduced estimates of persistent inward currents combined with reduced common synaptic inputs alongside poorer force control ability in the aged human VL, a muscle with established susceptibility for age-related functional losses. Though no significant age-related difference in MU discharge rates was observed, our findings highlight important aspects of impaired neural input to older muscles that likely contribute to functional decrements.

A smaller cross-sectional area of VL and a lower knee extensor muscle strength were observed in older participants, which is consistent with the wider ageing literature [8, 45]. However, the lack of a statistically significant difference in MU discharge rates is not aligned with previous findings in this muscle using needle EMG recordings at the same sustained contraction intensity [8]. This may reflect differences in the population of MUs sampled, with needle EMG sampling from a greater depth of muscle than surface-based HD EMG used here [46]. Additionally, this may be due to the slightly lower mean age of the older group (67.2 ± 8.9 vs 71.4 ± 6.2 years) in the current study. Age-related reduction of VL MU discharge rate was also reported in ramped contractions [47].

Age-related differences in force control ability were only observed during the ramp and not the steady sustained contraction, highlighting the role of task complexity in age-related impairments [48]. Numerous factors associated with advancing age may also explain this poorer force control in a more complex contraction, including MU remodelling, reduced excitability of the MUs, decreased muscle spindle sensitivity, fewer common synaptic inputs, and/or impaired cutaneous afferents [49, 50]. Estimates of MU number in the VL are reduced in older age [8, 51], and although direct human evidence is lacking, animal models show large MUs are preferentially lost with ageing and denervated fibres are reinnervated by early recruited MUs [52], resulting in expansion of those earlier recruited MUs [53] such as those sampled in the current study (< 20% MVC). These MUs with a larger number of fibres, when activated, would have a greater influence on the force during their progressive re/derecruitment processes, and may partly explain the age-related differences of force control observed here. The progressive increase and decrease in force during the targeted isometric ramped contraction used here is partly reliant on afferent neuromodulatory feedback from the site of force application [54]. Several studies have reported a decrease of grey matter and white matter throughout the motor cortex with increasing age [55–57] and a strong correlation between cortical atrophy and fine control capacity [58]. Moreover, the process of afferent feedback from sensory receptors to spinal motoneurons triggers rapid responses in muscles, but these responses tend to decrease with age [59]. As the complexity of tasks increases, there is a corresponding rise in the common neural inputs to agonist–antagonist muscle pairs [60], and although not definitively proven, these effects may be amplified in older age [61]. Additionally, both animal and human studies have revealed a decreased proprioception in aged muscles [62] with the evidence of declined muscle spindle numbers and degeneration of the sensory nerve terminals, leading to the morphological adaptations at a peripheral level and modulation of mechanoreceptor gain at a central level [63, 64].

Although not directly assessed, the points made thus far are supported by the intramuscular coherence findings during the ramp contraction. Coherence in the alpha band (5–12 Hz) is associated with Ia afferent feedback [65] and when exploring the age-related differences, a significantly lower coherence estimate in the alpha band was observed in old only during the ramp contraction, corresponding to the poorer force tracking ability. This result supports evidence from previous studies, showing a greater loss of Ia afferent feedback [66] and an impaired ability to modulate Ia presynaptic inhibition [67] in older participants. Moreover, it has also been suggested that age-related deterioration of cutaneous afferent inputs may also contribute to reduced force control ability [68]. Therefore, a decreased ability to regulate the progressive increase and decrease in force may also be a result of the combination of reduced Ia afferent feedback from muscle spindles and reduced cutaneous afferents.

The effect of ageing on MU recruitment thresholds is inconclusive, or more accurately, it appears to be muscle-specific. In a single study of the biceps and triceps, the identified MUs were recruited earlier in older triceps, but not the biceps [22]. This study of VL found no age-related difference in recruitment or derecruitment threshold of the identified MUs, which was approximately 10% of MVC in both young and older participants during contractions up to 20% of MVC.

Compared to younger participants, we report a significantly reduced *ΔF* in the VL of older participants, supportive of studies of other muscles in the upper [22] and lower [23] limbs. However, the methods applied thus far are unable to determine the relative influence of facilitation and inhibition on this age-related difference. Serotonin and norepinephrine influence the magnitude of PICs [69] and ageing is associated with decreased levels of serotonin receptors and transporters in the brain [70, 71] as well as impaired norepinephrine synthesis and secretion [72], which may result in a reduced monoaminergic neurotransmission and PIC facilitation. Counter to this hypothesis, *ΔF* values in the soleus were increased following a remote handgrip contraction and did so to a similar extent in young and old [73]. This suggests the PIC facilitation induced by the handgrip contraction, be it serotonergic drive or otherwise, was not impaired by ageing. The same study also used reciprocal inhibition of the soleus with tibialis anterior tendon vibration and found PIC reductions to be unaffected by age, yet vibration applied to the Achilles tendon reduced tibialis anterior PIC amplitude to a greater extent in young [73]. Collectively these data suggest inhibitory control of PICs is impaired in older age, but this impairment is muscle-specific [25].

### Strengths/limitations

To our knowledge, this is the first study concurrently estimating common synaptic inputs and persistent inward currents in young and older male VL, and age-related impairments are evident in each. However, there are several limitations. Firstly, the results reported here were from males only which may not directly translate to females, as decreases in estrogen levels may also contribute to the alterations in circulating serotonin and its receptor densities in postmenopausal women [74], and *ΔF* of lower limb muscles is higher in young females than young males [75]. Secondly, the MU data we report are relevant to those recruited at lower contraction levels (< 25% MVC) and reveal little of those higher recruitment threshold MUs recruited at higher force levels, which would occur if absolute, rather than normalised forces were used. Thirdly, the right leg was uniformly assessed across all participants, so it is not possible to infer any bilateral differences that could contribute to the age-related neural impairments.

## Conclusion

The reduced muscle strength and control ability observed in older males is partially attributable to reduced estimates of persistent inward currents and common synaptic input, which occur independently of reductions in MU discharge rates. These findings have important implications in the field of healthy human ageing and should be considered when applying interventions to mitigate age-related functional decrements.

## Data Availability

The datasets generated and analysed during the current study are available from the corresponding author upon reasonable request.
